# Lessons from a multicentre retrospective study of peptide receptor radionuclide therapy combined with lanreotide for neuroendocrine tumours: a need for standardised practice

**DOI:** 10.1007/s00259-020-04712-2

**Published:** 2020-02-15

**Authors:** Vikas Prasad, Raj Srirajaskanthan, Christos Toumpanakis, Chiara Maria Grana, Sergio Baldari, Tahir Shah, Angela Lamarca, Frédéric Courbon, Klemens Scheidhauer, Eric Baudin, Xuan-Mai Truong Thanh, Aude Houchard, Clarisse Dromain, Lisa Bodei

**Affiliations:** 1 Department of Nuclear Medicine, University Hospital of Ulm, Albert-Einstein-Allee 23, Ulm, Baden-Württemberg Germany; 2 King’s College Hospital, London, UK; 3 Neuroendocrine Tumour Unit, Royal Free Hospital, London, UK; 4 European Institute of Oncology, Milan, Italy; 5 University of Messina, Messina, Italy; 6 Queen Elizabeth Hospital Birmingham, Birmingham, UK; 7 The Christie NHS Foundation Trust, University of Manchester, Manchester, UK; 8 IUCT-Oncopole, Toulouse, France; 9 Technical University Muenchen, Klinikum r.d. Isar, Munich, Germany; 10 Gustave Roussy, Villejuif, France; 11 Ipsen, Boulogne-Billancourt, France; 12 Department of Diagnostic and Interventional Radiology, CHUV University Hospital, Lausanne, Switzerland; 13 Molecular Imaging and Therapy Service, Department of Radiology, Memorial Sloan Kettering Cancer Center, New York, NY USA

**Keywords:** Peptide receptor radionuclide therapy, ^177^Lu-DOTATOC, ^177^Lu-DOTATATE, Lanreotide, Neuroendocrine tumours

## Abstract

**Purpose:**

PRELUDE aimed to assess use and effectiveness/safety of lanreotide autogel/depot (LAN) combined with ^177^Lu-DOTATOC or ^177^Lu-DOTATATE (LAN–peptide receptor radionuclide therapy [PRRT]) in patients with progressive neuroendocrine tumours (NETs).

**Methods:**

International, non-interventional, retrospective, non-comparative analysis of medical records from patients with progressive metastatic or locally advanced grade 1 or 2 gastroenteropancreatic (GEP)- or lung-NETs. The primary endpoint was progression-free survival (PFS) at end of last LAN–PRRT cycle. Secondary endpoints included PFS at last available follow-up, best overall response, objective response rate (ORR), presence and severity of diarrhoea and flushing, and safety. Post-hoc analyses were conducted to determine pre-treatment tumour growth rate (TGR) cutoffs that best predicted the ORR during treatment.

**Results:**

Forty patients were enrolled (GEP-NETs, *n* = 39; lung-NETs, *n* = 1). PFS rates were 91.7% at end of last LAN–PRRT cycle and 95.0% at last available follow-up. In the full analysis set, best overall response among patients with GEP-NETs (*n* = 23) was stable disease (*n* = 14, 60.9%), partial response (*n* = 8, 34.8%) and progressive disease (*n* = 1, 4.3%). The ORR was 27.3% at end of last LAN–PRRT cycle and 36.8% at last available follow-up. Optimal baseline TGR cutoffs for predicting ORR at these time points were 1.18% and 0.33%, respectively. At baseline, 81.0% of patients had diarrhoea or flushing; both remained stable or improved in most cases. No increased adverse drug reactions were reported.

**Conclusion:**

Despite the major recruitment shortfall for the PRELUDE study, effectiveness data were encouraging in this selected population, highlighting the potential usefulness and feasibility of LAN combined with and after PRRT in patients with GEP-NETs. The study also identified challenges associated with evaluating clinical practice in a rare-disease setting and highlighted the need for standardisation of PRRT procedures.

**Trial registration:**

Trial number: NCT02788578; URL: https://clinicaltrials.gov/ct2/show/NCT02788578

## Introduction

Neuroendocrine tumour (NET) heterogeneity poses significant challenges for management [1]. Recommended treatment options frequently target one specific pathway, often resulting in eventual escape from treatment response, such that subsequent treatment lines must address even more heterogeneous clones [2, 3]. This highlights the need for new strategies that combine drugs with different mechanisms. Somatostatin analogues (SSAs) are considered first-line systemic medical therapy by the European Neuroendocrine Tumor Society (ENETS) and in the National Comprehensive Cancer Network (NCCN) guidelines [4–6] and are an established anti-tumour therapy for advanced locoregional disease and/or distant metastatic gastroenteropancreatic (GEP)- and lung-NETs. They target somatostatin receptors (SSTR) on NETs, exerting their anti-tumour effect through several pathways and ultimately leading to tumour-cell apoptosis [7]. Peptide receptor radionuclide therapy (PRRT), an established treatment for well-differentiated metastatic NETs, also uses SSTR overexpression on NETs to deliver a high radiation dose using an SSA radiolabelled with a β-particle-emitting radioisotope [6]. PRRT delays progression of metastatic NETs and controls hormonal symptoms in functional NETs [8–10]. It is recommended by ENETS for grade 1/2 midgut NETs following medical therapy failure [5] and by the NCCN for progressive NETs following SSA therapy failure [6], and is being considered for lung-NETs that strongly express SSTRs [4, 5].

Anti-tumour benefits of combined PRRT–SSA therapy in patients with midgut NETs progressing under octreotide LAR (30 mg) were demonstrated in the NETTER-1 study [8]. Combination of ^177^Lu-DOTATATE and octreotide LAR (30 mg) resulted in longer progression-free survival (PFS) and higher response rate, versus high-dose octreotide LAR monotherapy (60 mg). Consequently, ^177^Lu-DOTATATE was approved in Europe and the USA for SSTR-positive GEP-NETs [11, 12]. Data from ERASMUS, which examined the effects of ^177^Lu-DOTATATE in patients with bronchial or GEP-NETs [13, 14], extended the indication to include other NETs.

In the large randomised placebo-controlled CLARINET study, lanreotide autogel/depot (LAN) demonstrated anti-tumour benefits in patients with metastatic GEP-NETs and predominantly stable disease (SD) [15]. Similar benefits were observed in patients with progressive GEP-NETs in the CLARINET open-label extension and in a single-arm, open-label study [16, 17]. However, no data were available on the efficacy and safety of combined PRRT–LAN. The aim of the PRELUDE (**P**eptide **RE**ceptor radionuclide therapy in combination with **L**anreotide a**U**togel/depot in progressive **D**igestive and bronchopulmonary n**E**uroendocrine tumours) study was therefore to describe the use and investigate the effectiveness and safety of LAN combined with ^177^Lu-DOTATOC or ^177^Lu-DOTATATE in patients with progressive GEP- and lung-NETs. Also, at the time of study design, there were no data reporting the effects of SSA combined with, or for maintenance after, PRRT in a broader NET population (NETTER-1 recruited only patients with midgut NETs). Thus, there was a need to determine how LAN–PRRT was used in clinical practice (e.g. in which tumours, at what stage, at what doses/dosing intervals) and whether the combination treatment was beneficial. In addition, recognising the limitations of response evaluation criteria in solid tumours (RECIST) as a tool for assessing response to PRRT in slow-growing NETs, we performed a post-hoc analysis of tumour growth rate (TGR) measured using computed tomography (CT) or magnetic resonance imaging (MRI) to further assess the putative anti-tumour benefits of PRRT–LAN.

## Materials and methods

### Study design

**Fig. 1:**
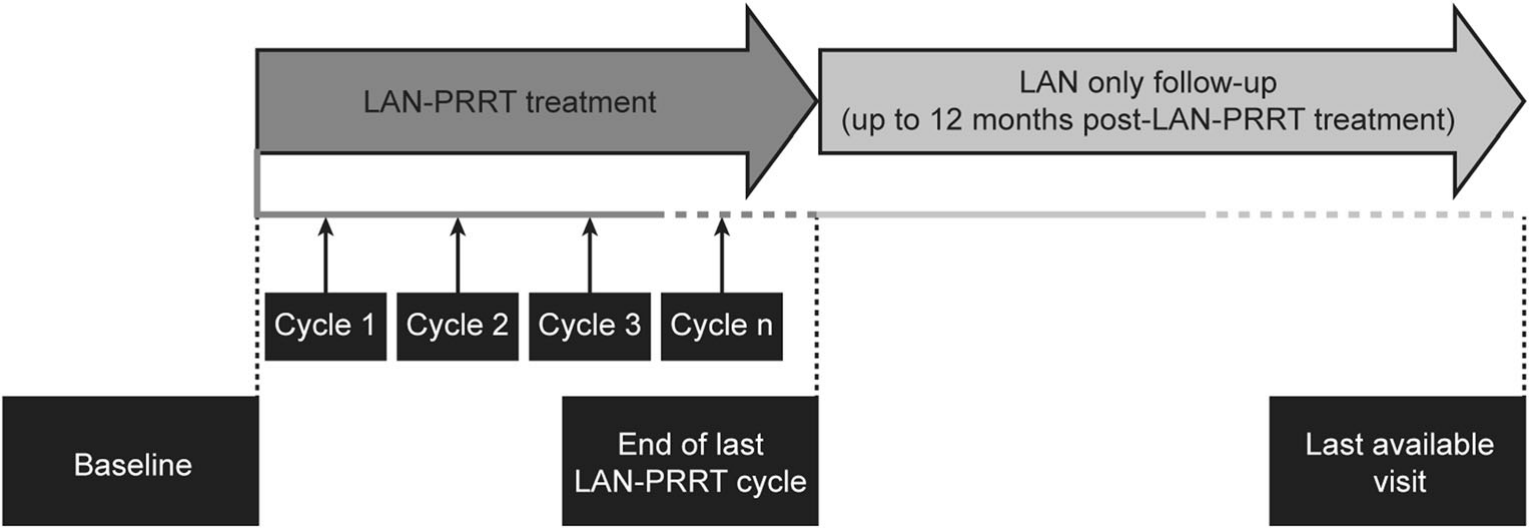
Study design. LAN–PRRT, lanreotide autogel/depot combined with peptide receptor radionuclide therapy

An international, non-interventional, retrospective, non-comparative analysis of the medical records of patients receiving LAN administered with ^177^Lu-DOTATOC or ^177^Lu-DOTATATE (NCT02788578; Fig. 1). Fifty centres across 12 countries were contacted initially. The original 6-month recruitment period (June–December 2016) was extended to > 13 months (to July 2017) to enhance enrolment.

### Patients

Patients (≥ 18 years) with metastatic or locally advanced grade 1 or 2, SSTR-positive (grade ≥ 2 on Krenning or positron emission tomography [PET] modified Krenning scale) primary GEP- or lung-NETs were enrolled. Other inclusion criteria were radiologically documented PD with evaluable imaging (CT or MRI scans) performed within 12 months and within 6 months before the first LAN–PRRT cycle; ≥ 1 prior injection of LAN in the 8 weeks before the first LAN–PRRT cycle; ^177^Lu-DOTATOC or ^177^Lu-DOTATATE total cumulative activity of ≥ 500 mCi (18.5 GBq); continual LAN throughout LAN–PRRT combination cycles.

Exclusion criteria were missing data on LAN treatment or cumulative activity of ^177^Lu-DOTATOC or ^177^Lu-DOTATATE; previous PRRT; and CT/MRI imaging not performed at the end of the last LAN–PRRT cycle. Patients were followed for up to 12 months after the last LAN–PRRT cycle.

This study was conducted in accordance with the Declaration of Helsinki, international ethical guidelines for epidemiological studies, Proper Conduct in Epidemiologic Research, Good Pharmacoepidemiology Practice, Good Pharmacovigilance Practice, local regulatory requirements and local routine medical practice. All patients (or family members/representatives) signed an informed consent form before enrolment, allowing their records to be included. The study protocol and amendments, patient information leaflet and consent form were reviewed and approved by an Independent Ethics Committee (IEC)/Institutional Review Board (IRB) before the start of the study in all countries in which the study was conducted (see Online Resource Table 1 for IECs/IRBs).

**Table 1:** Patient disposition and reasons for exclusion (enrolled population)

Population	GEP-NET (*N* = 39)	All patients (*N* = 40)
FAS, *n* (%)	23 (59)	24 (60)
Reason for exclusion: missing data for CT or MRI at the end of the last LAN–PRRT cycle	16 (41)	16 (40)
PP population*, *n* (%)	0	0
Reasons for exclusion:		
Not included in FAS	16 (41)	16 (40)
Eligibility criteria violation^†^	23 (59)	24 (60)
Time window violation^‡^	1 (2.6)	1 (2.5)
Safety population, *n* (%)	30 (76.9)	31 (77.5)
Reason for exclusion:no documented LAN–PRRT cycle	9 (23.1)	9 (22.5)

*Excluded patients could have more than one reason for exclusion. ^†^All patients, except one, had an eligibility criteria violation: radiologically documented PD in the year preceding initiation of the LAN–PRRT combination therapy (one patient was incorrectly assigned as having this eligibility criteria violation). ^‡^One patient had a time window violation: the CT/MRI scan was performed 1.5 months before the end date of the last LAN–PRRT cycle

*CT*, computed tomography; *FAS*, full analysis set; *GEP-NET*, gastroenteropancreatic neuroendocrine tumour; *LAN*, lanreotide autogel/depot; *MRI*, magnetic resonance imaging; *PD*, progressive disease; *PP*, per protocol; *PRRT*, peptide receptor radionuclide therapy

### Treatment

No investigational products were administered prospectively; only data on previous LAN and LAN–PRRT administration were collected. LAN injections were performed continuously throughout the LAN–PRRT combination cycles (≥ 1 LAN injection between each PRRT cycle). LAN was administered by deep subcutaneous injection, with injection frequency determined by investigators according to hospital clinical practice; PRRT (^177^Lu-DOTATOC or ^177^Lu-DOTATATE) was infused intravenously. Treatment decisions were made by investigators before enrolment according to local routine practices. Patients with prior and concomitant therapies for NETs (including surgery, molecular targeted therapy, chemotherapy and SSAs) were not excluded.

### Assessments and endpoints

#### Assessments

Data were collected at baseline (before administration of treatment on day 1 of first LAN–PRRT cycle), after the last LAN–PRRT cycle, and at the last available follow-up visit up to 12 months post-treatment. Digital/printed copies of radiological images from study centres were sent to an independent radiologist for central assessment according to RECIST v1.1. Disease progression status at baseline was assessed by central review (using MRI/CT scans performed within 12 months and 6 months before study baseline).

Baseline disease characteristics included World Health Organization (WHO) tumour grade and Ki67 proliferative index, tumour uptake score (based on Krenning score or PET-modified Krenning score), quality of hepatic SSTR expression (centrally assessed on target lesions on liver metastasis only), number of liver and bone metastases and hepatic tumour load. Quality of SSTR expression measured the heterogeneity of target lesions; patients with ≥ 50% and < 50% heterogeneous target lesions were defined as having heterogeneous expression (different levels of uptake) and homogeneous expression, respectively.

#### Effectiveness endpoints

Primary effectiveness endpoint was PFS rate (centrally assessed, RECIST v1.1) in patients with GEP- and lung-NETs at the end of the last LAN–PRRT cycle. Secondary effectiveness endpoints included PFS rate at last available follow-up visit, best overall response (OR), objective response rate (ORR) at the end of the last LAN–PRRT cycle and at last available follow-up, change from baseline in the presence and severity of diarrhoea and flushing at the end of the last LAN–PRRT cycle and at last available follow-up, and change from baseline in the tumour biomarker chromogranin A (CgA) at the end of the last LAN–PRRT cycle.

Two post-hoc analyses were performed as follows: (1) TGR, assessed using the MRI and CT scans taken at the following time points: within 12 months and 6 months before start of treatment, between start of treatment and end of last LAN–PRRT cycle (i.e. within 6 months before start of treatment and end of last LAN–PRRT cycle), and between end of last LAN–PRRT cycle and last available follow-up; (2) pre-treatment TGR cutoffs that best predicted ORR at the two subsequent time points in the study (i.e. end of last LAN–PRRT cycle and last available follow-up).

#### Safety endpoints

Secondary safety endpoints included change in body weight from baseline to the end of the last LAN–PRRT cycle and at the last available follow-up visit; incidence of nephro-, haemato- and hepatotoxicity events (using Common Terminology Criteria for Adverse Events v4.0) at baseline, at the end of the last LAN–PRRT cycle and at last available follow-up; and incidence of vomiting during infusion at the end of the last LAN–PRRT cycle.

### Statistical analyses

Assuming a sample size of 150 patients, a two-sided 95% confidence interval (CI) for a sample proportion using the normal approximation extend 8% from the observed proportion for an expected proportion of 50%. Statistical analysis was performed using Statistical Analysis System (SAS^®^) version 9.4 (SAS Institute Incorporated, Cary, NC, USA).

Statistical analyses for all effectiveness and safety endpoints were descriptive only; no statistical testing was performed. Descriptive summary statistics included number of documented data, mean and standard deviation (SD). Two-sided 95% CI (using Wilson’s score without continuity correction) were calculated for every relevant proportion, mean and median.

The primary effectiveness endpoint was defined as the rate of patients still alive and with no disease progression at the end of the last LAN–PRRT cycle. Analyses of PFS rates were evaluated from Kaplan–Meier estimates with two-sided 95% CIs. PFS, best OR and ORR were assessed centrally (RECIST v1.1). Presence/absence and severity of diarrhoea and/or flushing were reported using descriptive qualitative statistics, including 95% CIs. Quantitative statistics were used for change from baseline in body weight and in the tumour biomarker CgA (with the upper limit of normal presented using descriptive quantitative statistics). Incidence and grade of nephro-, haemato- and hepatotoxicity events and vomiting during each infusion were presented using descriptive qualitative statistics, including 95% CIs.

TGR—expressed as the percentage increase in tumour volume during 1 month—was calculated from the sum of the longest diameter of target lesions [18–20] between the two (centrally read) MRI/CT scans taken at each time point. TGR thresholds for predicting ORR were derived from receiver operating characteristic (ROC) curves with maximisation of the Youden index.

Four different analysis populations were defined as follows: enrolled (all consenting patients who were fully informed about the study before data collection), full analysis set (FAS) (all enrolled patients with ≥ 1 measurable lesion at baseline and at the end of the last LAN–PRRT cycle), per protocol (PP) (all patients in the FAS who had no major protocol violations/deviations and who had progression at baseline, assessed centrally according to RECIST v1.1.) and safety (all enrolled patients with ≥ 1 cycle of LAN–PRRT documented). Effectiveness analyses were performed on the FAS and PP populations; safety analyses were performed on the safety population.

## Results

### Patients

**Fig. 2:**
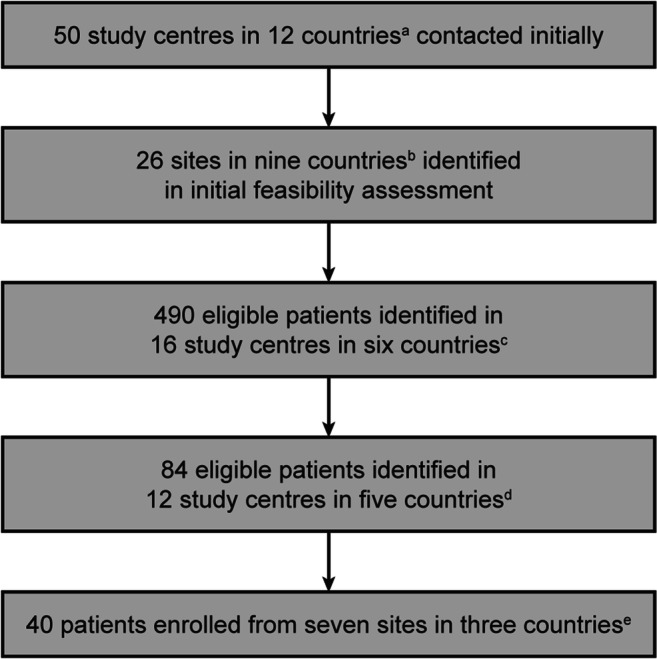
Study enrolment. ^a^Australia, Austria, Denmark, France, Germany, Italy, the Netherlands, Poland, Spain, Sweden, Switzerland, UK; ^b^Australia, Denmark, France, Germany, Italy, the Netherlands, Poland, Sweden, UK; ^c^Australia, France, Germany, Italy, the Netherlands, UK; ^d^Australia, France, Germany, Italy, UK; ^e^France, Italy, UK. Reasons for non-participation of study centres initially identified includes involvement in competing studies and overestimation of number of suitable patients initially identified

Following an initial feasibility assessment, 26 sites in nine countries were open to recruitment; ultimately, patient recruitment was terminated after enrolment of 40 patients from seven sites (see Fig. 2 for more details).

The main reasons underlying the recruitment shortfall were lack of proper documentation of LAN treatment during PRRT (as combination treatment was administered at a different location); use of different combinations of PRRT (^90^Y-DOTATATE and ^177^Lu-DOTATATE); use of single-photon emission CT or PET to assess NET progression; therapy with LAN–PRRT plus capecitabine; difficulties obtaining CT/MRI scan images (e.g. not centralised [retained at home]) and total cumulative activity of ^177^Lu < 500 mCi/18.5 GBq.

Among the 40 patients in the enrolled population, 39 had a GEP-NET, and one had a lung-NET. The FAS comprised 24 patients (23 with GEP-NETs and one with a lung-NET). The main reasons for exclusion from the FAS were missing CT/MRI data at the end of the last LAN–PRRT cycle (Table 1).

Table 2 summarises patient demographics and LAN usage prior to LAN–PRRT in the enrolled population and FAS. In both cohorts, most patients with GEP-NETs were male, and more than half were aged ≤ 65 years. All other demographic parameters were similar between GEP-NET groups. The most common LAN dosage in the GEP-NET group was 120 mg every 28 days in both the enrolled (*n* = 16; 53.3%) and the FAS (*n* = 12; 52.2%) populations. Many patients with GEP-NETs (FAS) reported ≥ 1 important medical history related to NETs (47.8%), including abdominal pain (21.7%) and diarrhoea (26.1%).

**Table 2:** Patient demographics and prior LAN dose/frequency (enrolled and FAS populations)

Demographic		Enrolled population	FAS population		
		GEP-NET (*N* = 39)	All patients (*N* = 40)	GEP-NET *(N* = 23)	ALL PATIENTS (*N* = 24)
Sex, *n* (%)	Male	28 (71.8)	29 (72.5)	15 (65.2)	16 (66.7)
Age (years)	Mean (SD)	62.4 (11.8)	62.6 (11.7)	60.0 (12.0)	60.5 (12.0)
	Median (range)	64 (34–80)	64 (34–80)	59.0 (34–80)	61.0 (34–80)
Age (years) in classes, *n* (%)	≤ 65	21 (53.8)	21 (52.5)	15 (65.2)	15 (62.5)
	> 65	18 (46.2)	19 (47.5)	8 (34.8)	9 (37.5)
Height (cm)	Mean (SD)	167.8 (11.3)	168.5 (11.7)	168.3 (12.4)	169.3 (12.7)
	Median (range)	169.0 (144–193)	169.5 (144–193)	169.0 (144–193)	169.5 (144–193)
Body mass index (kg/m^2^)	Missing	12	12	4	4
	Mean (SD)	26.49 (6.00)	26.53 (5.89)	27.1 (5.5)	27.1 (5.4)
	Median (range)	25.92 (16.9–41.5)	26.14 (16.9–41.5)	26.4 (18.9–41.5)	26.6 (18.9–41.5)
Body mass index (kg/m^2^) class, n (%)	Missing	12	12	4	4
	< 18	1 (3.7)	1 (3.6)	–	–
	≥ 18 and ≤ 25	11 (40.7)	11 (39.3)	8 (42.1)	8 (40.0)
	> 25	15 (55.6)	16 (57.1%)	11 (57.9)	12 (60.0)
Last dose/frequency of injection of LAN prior to the first LAN—PRRT cycle, *n* (%)	*n*	30	31	23	24
	Missing data	9	9	0	0
	120 mg every 21 days	5 (16.7)	5 (16.1)	5 (21.7)	5 (20.8)
	120 mg every 28 days	16 (53.3)	16 (51.6)	12 (52.2)	12 (50.0)
	60 mg every 28 days	3 (10.0)	3 (9.7)	3 (13.0)	3 (12.5)
	90 mg every 28 days	4 (13.3)	5 (16.1)	2 (8.7)	3 (12.5)
	Other	2 (6.7)	2 (6.5)	1 (4.3)	1 (4.2)

The denominator for the percentage was based on the patients with available responses

Nine patients had missing data for the prior LAN administration: these nine patients corresponded to the nine patients excluded from the safety population

*FAS*, full analysis set; *GEP-NET*, gastroenteropancreatic neuroendocrine tumour; *LAN*, lanreotide autogel/depot; *PRRT*, peptide receptor radionuclide therapy; *SD*, standard deviation

Baseline tumour characteristics are presented in Table 3. In the GEP-NET enrolled and FAS populations, mean (SD) time since tumour diagnosis was approximately 4 years, and primary tumours were located predominantly in the ileum, although the origin was unknown in one-quarter to one-third of patients. Hepatic tumour load was ≤ 25% in > 80% of patients and almost all patients presented with ≥ 1 liver metastasis; only a limited number had bone metastases.

**Table 3:** Baseline tumour characteristics (enrolled and FAS populations)

Characteristics	Enrolled population		FAS population	
	GEP-NET (*N* = 39)	All patients (*N* = 40)	GEP-NET (*N* = 23)	All patients (*N* = 24)
Mean (SD) time from initial diagnosis, years	3.7 (3.1)	3.7 (3.1)	3.9 (3.3)	3.9 (3.2)
Location of primary tumour for GEP-NET, *n* (%)				
Colon, right	8 (20.5)	8 (20.5)	2 (8.7)	2 (8.7)
Colon, sigmoid	1 (2.6)	1 (2.6)	1 (4.3)	1 (4.3)
Ileum	13 (33.3)	13 (33.3)	8 (34.8)	8 (34.8)
Pancreas	4 (10.3)	4 (10.3)	3 (13.0)	3 (13.0)
Rectum	1 (2.6)	1 (2.6)	1 (4.3)	1 (4.3)
Stomach	2 (5.1)	2 (5.1)	0	0
Unknown	10 (25.6)	10 (25.6)	8 (34.8)	8 (34.8)
Tumour grade, *n* (%)				
Grade 1	21 (53.8)	21 (52.5)	12 (52.2)	12 (50.0)
Grade 2	17 (43.6)	18 (45.0)	11 (47.8)	12 (50.0)
Grade 3	1* (2.6)	1* (2.5)	0	0
Proliferation index Ki67 (%), *n* (%)	32	33	18	19
Missing	7	7	5	5
≤ 2	15 (46.9)	16 (48.5)	7 (38.9)	8 (42.1)
> 2 and ≤ 20	17 (53.1)	17 (51.5)	11 (61.1)	11 (57.9)
Presence of liver metastases, *n* (%)				
Yes	37 (94.9)	38 (95.0)	23 (100.0)	24 (100.0)
Presence of bone metastases, *n* (%)				
Missing	2	2	2	2
Yes	4 (10.8)	4 (10.5)	3 (14.3)	3 (13.6)
Global overall Krenning scale centrally assessed, *n* (%)	27	28	22	23
Missing	12	12	1	1
Grade 2	4 (14.8)	4 (14.3)	4 (18.2)	4 (17.4)
Grade 3	4 (14.8)	4 (14.3)	4 (18.2)	4 (17.4)
Grade 4	19 (70.4)	20 (71.4)	14 (63.6)	15 (65.2)
Global heterogeneity in tumour uptake centrally assessed, *n* (%)	24	25	19	20
Missing	15	15	4	4
Yes	9 (37.5)	9 (36.0)	8 (42.1)	8 (40.0)
No	15 (62.5)	16 (64.0)	11 (57.9)	12 (60.0)
Scale used to assess performance status, *n* (%)	23	24	15	16
Missing	16	16	8	8
ECOG performance status	18 (78.3)	19 (79.2)	10 (66.7)	11 (68.8)
≤ 2	17 (94.4)	18 (94.7)	10 (100.0)	11 (100.0)
> 2	1 (5.6)	1 (5.3)	0	0
Karnofsky performance status	5 (21.7)	5 (20.8)	5 (33.3)	5 (31.3)
≥ 60	5 (100.0)	5 (100.0)	5 (100.0)	5 (100.0)
Hepatic tumour load, *n* (%)	30	31	23	24
Missing	9	9	0	0
≤ 25	26 (86.7)	27 (87.1)	19 (82.6)	20 (83.3)
> 25	4 (13.3)	4 (12.9)	4 (17.4)	4 (16.7)
Progression before baseline^†^ (as per RECIST centrally assessed) [a], *n* (%)	30	31	23	24
Missing	9	9	0	0
Yes	1 (3.3)	1 (3.2)	1 (4.3)	1 (4.2)
No	29 (96.7)	30 (96.8)	22 (95.7)	23 (95.8)

*Patient did not meet the inclusion criterion (grade 1 or 2 tumour); however, as this was considered a minor protocol deviation, the patient’s data were included in the analyses

^†^Progression at baseline based on scans performed within 12 months and within 6 months prior to the first LAN–PRRT cycle. The denominator for the percentage was based on the patients with available responses. One patient reported lung localisation of the primary tumour (lung-NET)

*ECOG*, Eastern Cooperative Oncology Group; *FAS*, full analysis set; *GEP-NET*, gastroenteropancreatic neuroendocrine tumour; *LAN*, lanreotide autogel/depot; *Max*, maximum; *Min*, minimum; *PRRT*, peptide receptor radionuclide therapy; *RECIST*, response evaluation criteria in solid tumours; *SD*, standard deviation

### Study drug administration

In the safety population, median (range) duration of LAN exposure was 37.0 (16.7–90.0) months overall, 10.5 (0.7–61.7) months prior to LAN–PRRT, 14.2 (7.0–24.0) months during LAN–PRRT and 12.6 (6.1–32.5) months during LAN only follow-up. Median (range) cumulative administered activity of PRRT was 29.6 (21.2–31.7) GBq. Mean (95% CI) number of LAN–PRRT cycles was 4.4 (4.0, 4.9), with most patients (18/23, 78.3%) receiving ≤ 4 cycles. All patients received ^177^Lu-DOTATATE from cycles 1 to 8, except for one patient who received ^177^Lu-DOTATOC at cycle 2.

**Fig. 3:**
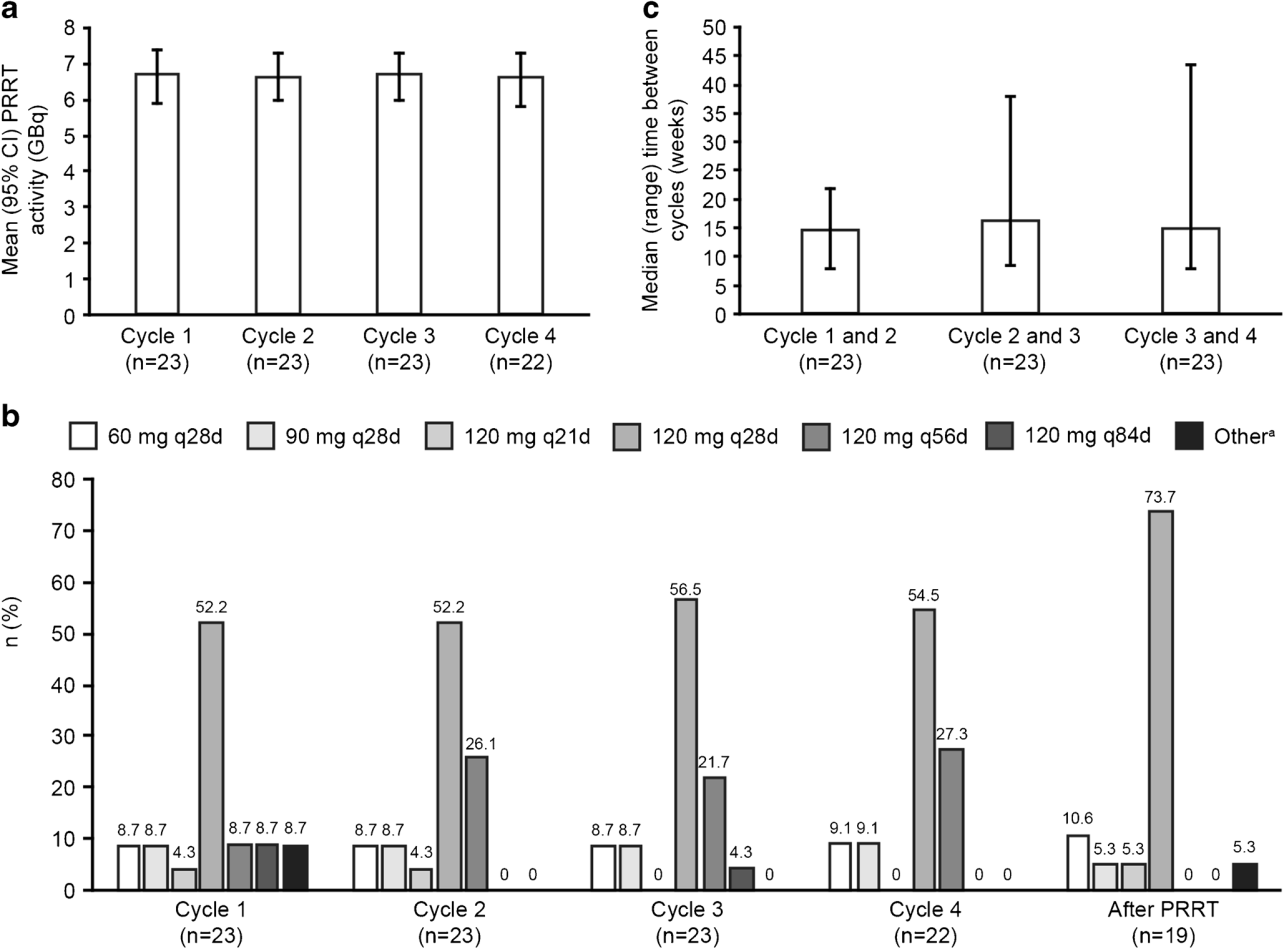
Study drug administration in patients with GEP-NETs (FAS populations). **a** PRRT activity, **b** LAN dose, **c** Interval between cycles. ^a^120 mg every 37 days (*n* = 2), dose not specified (*n* = 1). Note that some patients had up to 8 cycles of PRRT. CI, confidence interval; FAS, full analysis set; GEP-NET, gastroenteropancreatic neuroendocrine tumour; LAN, lanreotide Depot/Autogel; PRRT, peptide receptor radionuclide therapy; q21d, every 21 days; q28d, every 28 days; q56d, every 56 days; q84d, every 84 days

Mean (95% CI) activity of PRRT administered remained stable from cycle 1 to cycle 4 (Fig. 3a), consistent with the ^177^Lu-DOTATATE treatment regimen used in the NETTER-1 study and in line with its indication (i.e. four infusions of 7.4 GBq each) [8, 12]. All patients received a similar cumulative activity regardless of the number of cycles received; therefore, patients who received > 4 cycles received a lower dose of ^177^Lu-DOTATATE per cycle.

During cycles 1 to 4, LAN 120 mg was prescribed in > 80% of patients, and the LAN-dosing regimen was 120 mg every 28 days for ≥ 50% of patients, with most other patients receiving LAN 120 mg every 56 days; one patient received LAN 120 mg every 21 days during cycles 1 and 2 (Fig. 3b). Over 70% of patients with GEP-NETs were prescribed LAN (any dose) every 28 days from cycles 1 to 4. The median (range) time between two successive cycles was higher than the recommended 8 to 12 weeks, ranging from 14.7 (7.9–21.7) weeks between cycles 1 and 2 to 16.3 (8.4–37.9) weeks between cycles 2 and 3 (Fig. 3c).

During the 12-month follow-up period, LAN was administered to 19/23 (82.6%) patients with GEP-NETs, most of whom (14/19, 73.7%) received 120 mg every 28 days. In these 19 patients, LAN 120 mg (any frequency) was administered in 79% of patients, and LAN every 28 days (any dose) was administered in approximately 90% of patients (Fig. 3b).

### Effectiveness

**Fig. 4:**
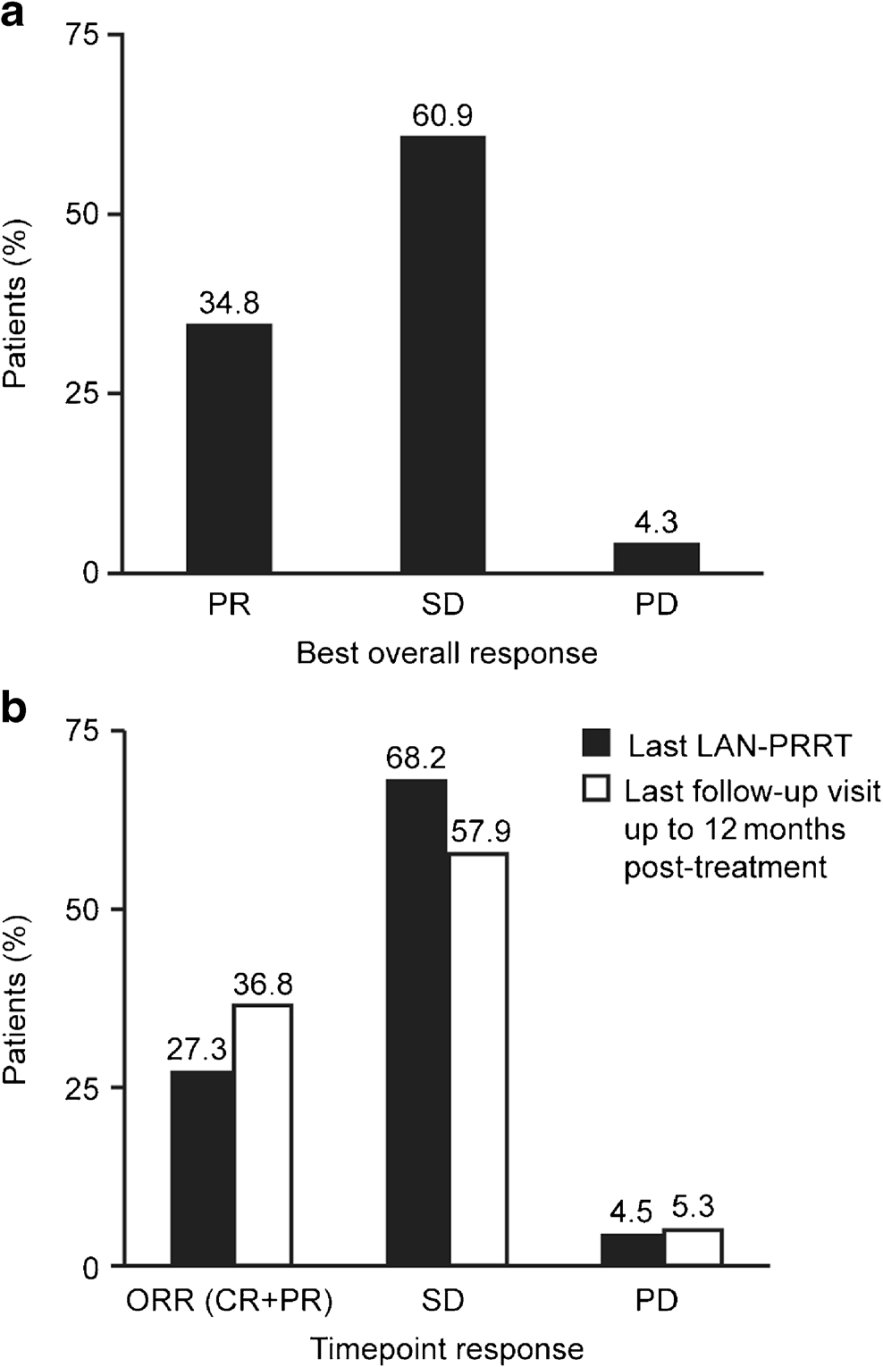
Best OR and time point response (FAS; patients with GEP-NETs). CR, complete response; FAS, full analysis set; GEP-NET, gastroenteropancreatic neuroendocrine tumour; OR, overall response; ORR, objective response rate; PD, progressive disease; PR, partial response; SD, stable disease

As only one patient had lung-NETs, all results in this section are described for patients with GEP-NETs only (*n* = 23, FAS). The median study duration was 22.64 months (95% CI 18.86, 25.54). The PFS rate (95% CI) was 91.7% (53.9, 98.8) at the end of the last LAN–PRRT cycle and 95.0% (69.5, 99.3) at last available follow-up. The best OR was SD in 14 (60.9%) patients, PR in eight (34.8%) patients and PD in one (4.3%) patient (progressed at 14.7 months, Fig. 4a). The patient with PD was a 45-year-old male, with a primary tumour of the ileum (grade 1) and liver metastases, and SD before treatment. One patient with SD at the end of the last LAN–PRRT cycle went on to achieve PR at last available follow-up (all other patients remained stable in their category). The ORR (95% CI) was 27.3% (13.2, 48.2) at the end of the last LAN–PRRT cycle and 36.8% (19.1, 59.0) at last available follow-up (Fig. 4b).

**Fig. 5:**
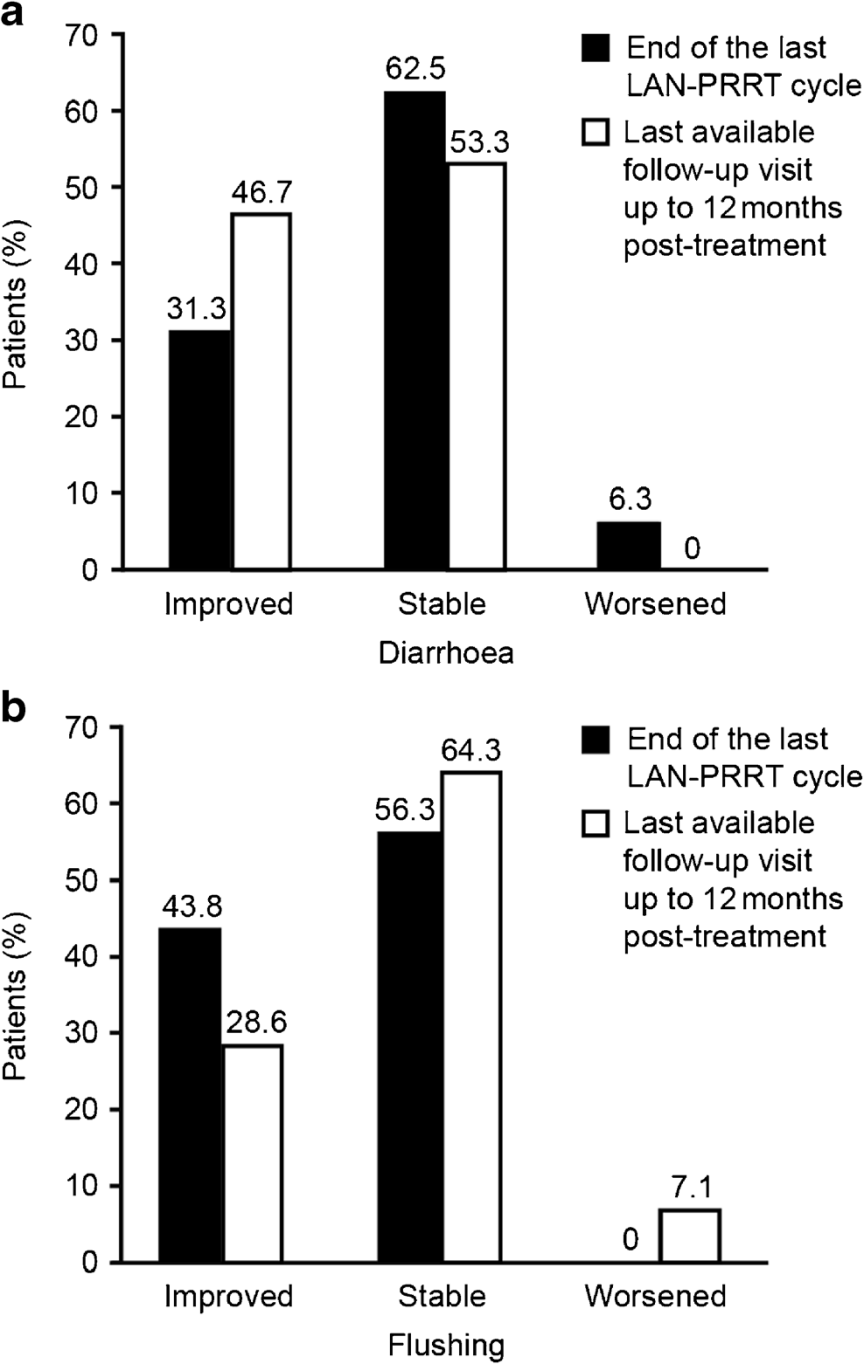
Change from baseline in diarrhoea or flushing (FAS; patients with GEP-NETs). FAS, full analysis set; GEP-NET, gastroenteropancreatic neuroendocrine tumour; LAN–PRRT, lanreotide autogel/depot combined with peptide receptor radionuclide therapy. Data were missing for change from baseline in diarrhoea (end of last LAN–PRRT cycle, *n* = 7; last available follow-up visit to 12 months post-treatment, *n* = 4) and change from baseline in flushing (end of last LAN–PRRT cycle, *n* = 7; last available follow-up visit to 12 months post-treatment, *n* = 5)

At baseline, 81% of patients were symptomatic, reporting diarrhoea or flushing. In most patients, both these symptoms remained stable or improved from baseline (Fig. 5).

Mean and median CgA levels increased between baseline and the end of the last LAN–PRRT cycle (Online Resource Table 2). However, the results were limited by poor assay accuracy and large variability.

**Fig. 6:**
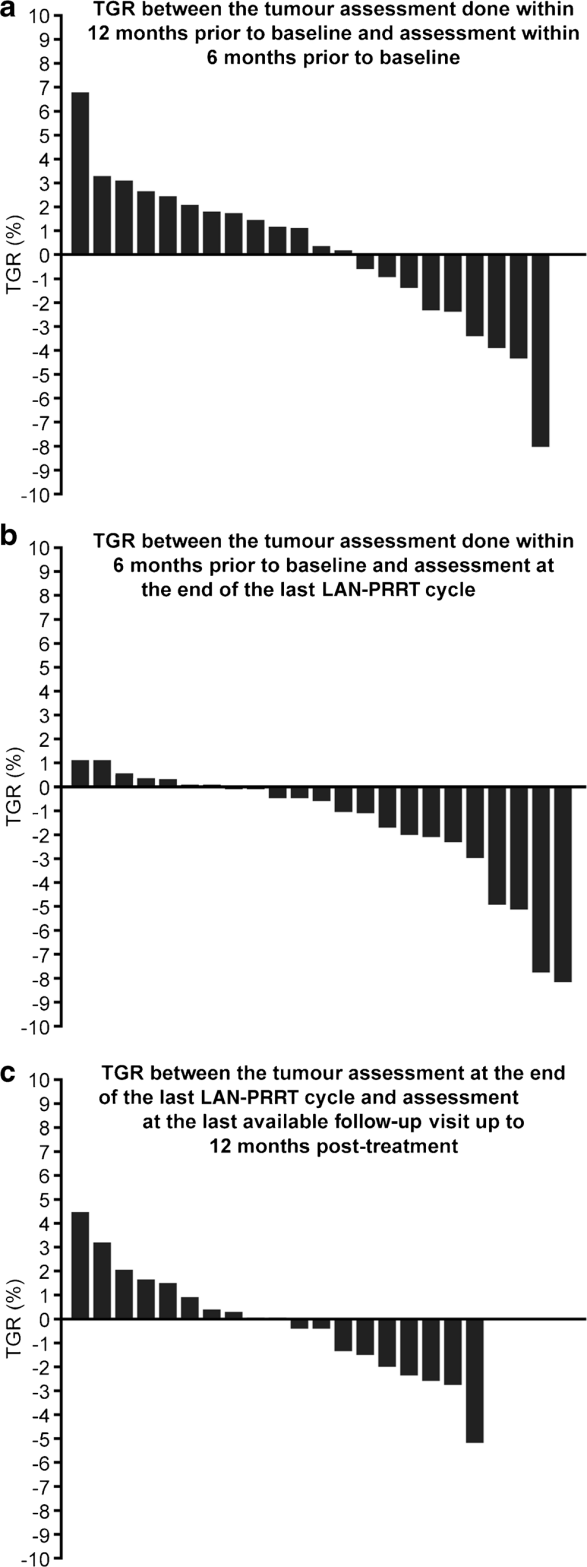
Tumour growth rate **a** before, **b** during and **c** after LAN–PRRT treatment (FAS; patients with GEP-NETs). FAS, full analysis set; GEP-NET, gastroenteropancreatic neuroendocrine tumour; LAN-ATG, lanreotide autogel-depot; LAN–PRRT, lanreotide autogel/depot combined with peptide receptor radionuclide therapy; TGR, tumour growth rate

Waterfall plots of patient TGRs (post-hoc analysis) showed individual progressions and regressions (Fig. 6). Mean (95% CI) TGR was 0.0% (− 1.4, 1.5) per month within 12 months and 6 months before the start of treatment, − 1.6% (− 2.7, − 0.4) per month between baseline and end of last LAN–PRRT cycle, and − 0.2% (− 1.3, 0.9) per month between the end of the last LAN–PRRT cycle and last available follow-up.

**Fig. 7:**
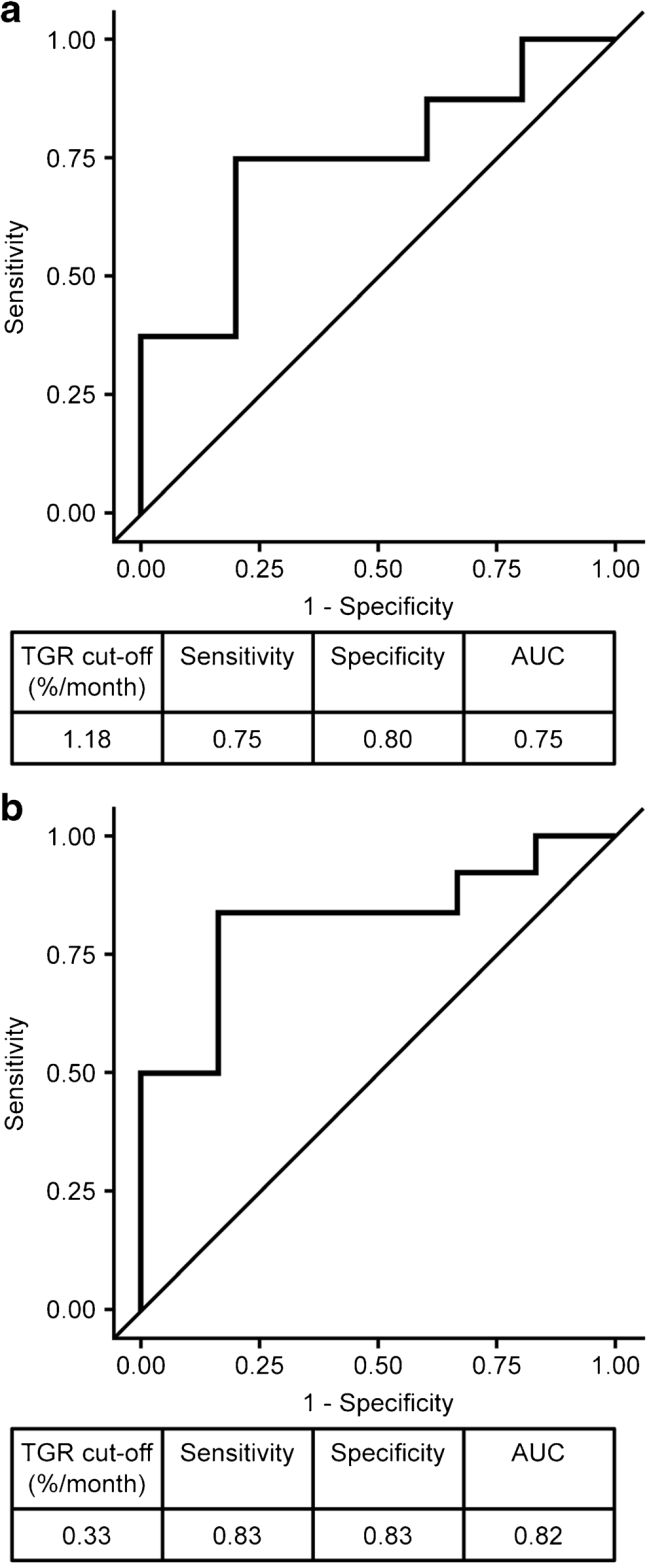
ROC analysis outputs determining pre-treatment TGR cutoffs for predicting ORR **a** at end of the last LAN–PRRT cycle and **b** at last available follow-up visit. AUC, area under the ROC curve; LAN–PRRT, lanreotide autogel/depot combined with peptide receptor radionuclide therapy; ORR, objective response rate; ROC, receiver operating characteristic; TGR, tumour growth rate

Optimal baseline TGR cutoff for predicting ORR at end of last LAN–PRRT was 1.18% (sensitivity 0.75, specificity 0.80, area under the plasma concentration–time curve [AUC] 0.75) (Fig. 7a), whereas optimal baseline TGR cutoff for predicting ORR at last available follow-up was 0.33% (sensitivity 0.83, specificity 0.83, AUC 0.82) (Fig. 7b).

### Safety

#### Adverse events

In the safety population (*n* = 31), 3 patients (9.7%) reported nephro-, haemato- or hepatotoxicity events, one during PRRT, one post-PRRT and one during both PRRT and post-PRRT. During LAN-PRRT treatment, the most frequently reported toxicity was haematotoxicity, occurring in two patients and contributing to 20/26 (76.9%) toxicity events. Haematotoxicity events were mainly grade 1 (12/20 events; 60.0%) with three grade 3 events (Table 4). No vomiting was reported during infusions, and no deaths occurred. No weight loss was reported, with a mean (95% CI) change in body weight of 0.5 (− 2.1, 3.0) kg between baseline and end of PRRT, and − 0.3 (− 4.7, 4.1) kg between baseline and last follow-up.

**Table 4:** Incidence of nephro-, haemato- and hepatotoxicity events (safety population)

Event	From day 1 of the first LAN–PRRT cycle to the end of the last LAN–PRRT cycle (*n* = 31)		From the end of the last LAN–PRRT cycle to the last available follow-up (*n* = 31)	
	*N*	*n* (%)	*N*	*n* (%)
Any events	26	2 (6.5)	5	2 (6.5)
Haematotoxicity/bone marrow toxicity	20	2 (6.5)	3	2 (6.5)
Grade 1	12	2 (6.5)	3	2 (6.5)
Grade 2	5	1 (3.2)	–	–
Grade 3	3	2 (6.5)	–	–
Hepatotoxicity	5	1 (3.2)	2	1 (3.2)
Grade 1	3	1 (3.2)	1	1 (3.2)
Grade 2	2	1 (3.2)	–	–
Grade 3	–	–	1	1 (3.2)
Renal toxicity	1	1 (3.2)	–	–
Grade 1	1	1 (3.2)	–	–

*LAN*, lanreotide autogel/depot; *n*, number of patients; *N*, number of events; *PRRT*, peptide receptor radionuclide therapy

## Discussion

The PRELUDE study aimed to describe the effectiveness of LAN combined with PRRT (LAN–PRRT) and as post-PRRT maintenance therapy in patients with progressive GEP- or lung-NETs. However, there was a major recruitment shortfall; only 40 patients were enrolled: 39 with GEP-NETs and one with lung-NETs. This reflects the challenges of conducting a retrospective study in a rare-disease setting, patients’ follow-up period occurring after the time of the analysis, and the strict inclusion/exclusion criteria, which many patients did not meet. In addition, CT/MRI scans were not always obtainable (e.g. retained by patients at home). Despite the recruitment shortfall, effectiveness data in the selected population with GEP-NETs were encouraging. High PFS rates were observed at the end of the last LAN–PRRT cycle (91.7%) and at last available follow-up, 12 months post-treatment (95.0%). Diarrhoea and flushing remained stable or were improved in most patients. Few toxicities were reported, and no new safety issues were identified, indicating that LAN–PRRT combination regimen had an acceptable safety/tolerability profile. These data add to the growing body of evidence supporting the use of SSA–PRRT in NETs, from the NETTER-1 study (*n* = 229) [8] and retrospective analysis conducted by Yordanova et al., which assessed ^177^Lu-octreotate plus octreotide LAR (median dose: 30 mg) or LAN (median dose: 120 mg) versus ^177^Lu-octreotate alone in 168 patients with GEP-NETs [21]. In these studies, there were significant improvements in PFS and response rates for SSA–PRRT compared with SSA alone [8] and PRRT alone [21]. However, it should be noted that certain aspects of the Yordanova study limit interpretation of the findings. For example, the authors used adapted WHO response criteria that have not been established for PRRT; as well as partial and complete responders, patients with SD and minimal response were also included in the clinical benefit rate and ORR, respectively; there were no detailed descriptions of PRRT cycle and SSA dose interval and no explanation regarding why some patients received SSAs whilst others did not [21]. PRELUDE also builds on the findings from the Yordanova study, by describing for the first time ORR (rather than best OR) at different time points (rather than at a single time point).

PRRT is a low-dose-rate irradiation intended to slow or stop tumour progression. Slower-growing tumours are significantly more likely to remain stable or shrink post-irradiation, suggesting that pre-treatment TGR may be predictive of the tumour response to PRRT [22, 23]. Indeed, post-hoc TGR analyses suggested tumour regression in some patients before, during and after LAN–PRRT and that ORR during or after LAN–PRRT was more likely if baseline TGR was ≤ 1.18%/month and ≤ 0.33%/month, respectively. Despite the initial TGR of 0% between pre-baseline and baseline in our study, a PR was still achieved in more than 30% of patients. However, these results should be interpreted with caution as TGR measurements were based on two target tumours in most patients; in the GREPONET study, which evaluated the value of TGR as a biomarker of outcome in 222 patients with NETs, the accuracy of TGR as a prognostic factor was found to be superior if four target lesions were used for TGR calculation [24]. Nevertheless, these findings suggest that the population enrolled into PRELUDE (i.e. patients with slowly progressing disease) are probably the best candidates for LAN − PRRT. It remains to be seen if TGR provides additional predictive benefit to other proposed and somewhat established parameters like the intensity of uptake and burden of disease [25].

Our findings that diarrhoea and flushing were stable or improved with LAN–PRRT agree with the symptomatic improvements reported following ^90^Y-edotreotide therapy in patients with carcinoid syndrome (CS) refractory to SSAs, and the known effectiveness of LAN for controlling CS symptoms [10, 26]. The clinical responses for diarrhoea and flushing continued to improve during treatment with LAN and during the follow-up period supporting the notion of maintenance therapy with LAN following PRRT. The adverse events reported were also consistent with published data on the use of PRRT [8, 27–29]. Most patients (52.2%) in the GEP-NET FAS group received a LAN dosage of 120 mg every 28 days. Across all cycles, 17 to 25% of patients in the FAS received a LAN dose < 120 mg, and 20 to 27% received LAN injections greater than 28 days apart. The reasons for the use of doses lower than 120 mg every 28 days were not recorded but might reflect caution on the part of clinicians when using LAN in combination with PRRT. Indeed, a much higher percentage of patients were prescribed 120 mg every 28 days during the follow-up period when patients were treated with LAN only. As there was no increase in reported adverse drug reactions compared with LAN-only treatment, we observe that LAN–PRRT had an acceptable safety/tolerability profile.

The study was associated with several limitations but nevertheless provides important learnings related to the management of NETs in clinical practice and the conduct of future clinical trials. One limitation of the current study was the assessments used. At baseline, most patients for whom data were available did not have confirmed PD (according to RECIST v1.1) when centrally assessed, despite this being an inclusion criterion, demonstrating a discrepancy between local and central assessment. This indicates that RECIST might not be used routinely in clinical practice; instead clinicians may evaluate other scan characteristics (e.g. tumour growth or volume of multiple lesions). In addition, the reason for initiating PRRT treatment may also relate to patients’ biological, physical or general condition. In the current study, over 80% of patients were symptomatic at baseline, which may have influenced investigators’ decisions to initiate a treatment with PRRT. Limitations associated with size-based evaluations (such as RECIST) are also recognised as they may underestimate the response to some treatments [30]. Additionally, the CgA results were limited by poor assay accuracy (high false-positive and false-negative rates) and large variability (as reported previously [31]); therefore, very little can be deduced from these findings. Encouragingly, since PRELUDE was initiated, several improvements in this therapy area have been made, with the NCCN updating its guidelines to align future studies in this field [6]. Use of ^177^Lu-DOTATATE has now been approved in Europe for adults with unresectable or metastatic, progressive, well-differentiated SSTR-positive GEP-NETs, providing clinicians with a clear posology and treatment schedule for PRRT [12].

Inherent limitations associated with retrospective studies, such as an increased risk for selection bias, necessarily apply to PRELUDE. In addition, there may have been under-reporting of treatment-related toxicities, AEs and other safety issues due to the retrospective nature of the study. However, PRELUDE was the first and only international multicentre retrospective study to use central assessment to measure PFS in a highly homogenous patient population receiving LAN–PRRT. Other limitations relate to the strict inclusion/exclusion criteria. In some cases, different combinations of PRRT (e.g. ^90^Y-DOTATATE and ^177^Lu-DOTATATE) or different doses of ^177^Lu-DOTATATE had been used, which was reflective of real practice. Patients in whom total cumulative activity of ^177^Lu was less than 500 mCi (18.5 GBq) also had to be excluded. In some geographies, SPECT and PET imaging had been routinely used to assess NET progression, rather than CT/MRI. Together, these findings highlight substantial differences across the community in terms of use and follow-up of non-approved treatments (LAN and PRRT in both combination and maintenance) and the lack of standardisation in nuclear medicine. The study may have been affected by ‘survivor bias’; data from deceased patients were less likely to be included because in some countries (Germany, France, UK), data collection was not allowed, investigators were reluctant to approach families to obtain consent to use the data, or authorities requested some rationale to do so. Furthermore, it was not always possible to obtain scans if results had been lost or sent to relatives.

At the time of the study, PRRT was not approved in this patient population and guidelines for practises and availability of PRRT, and standardisation of PRRT protocols were limited. Performing a retrospective study thus posed a major challenge. A lack of data sharing between centres meant diverse methods were adopted, leading to marked variability in results between centres. Disparities also arose owing to the use of PET and CT imaging and different acquisition protocols, leading to the realisation that there was no consensus on the timing for imaging assessment, imaging methods, or on how to manage the collection of CT/MRI scans. Differences in tumour assessment techniques also further limited the study. These results highlight a real need for standardisation of PRRT procedures in clinical practice.

Despite these limitations and the low number of patients enrolled, findings from PRELUDE remain robust and resonate with those reported in other studies. The low number of patients with bone metastases at baseline in this study, together with high PFS rate, concords with reported estimates of only 8 to 19% of bone metastases being associated with GEP-NETs [32–35], and that the absence of bone metastases is associated with a longer time to progression in patients receiving PRRT [36].

In conclusion, PRELUDE provides important information for clinicians managing patients with NETs, suggesting that LAN may be effective before, during and after PRRT in patients with metastatic or locally advanced GEP-NETs. Results of the post-hoc analyses also highlight the potential role of baseline TGR to assess and predict response to LAN–PRRT. The strict inclusion criteria in PRELUDE and use of central radiological assessment to measure PFS and ORR increased the robustness of these findings. Conducting such retrospective studies in the absence of standardised or approved treatment protocols is challenging but can provide important insights into how clinical practice and future study designs can be improved. As such, a prospective study of LAN–PRRT in a larger population of patients with GEP and lung NETs, using a standardised treatment protocol, is warranted.
